# Global Prevalence of Self‐Reported Alarm Features in the General Population and Among Individuals With Disorders of Gut–Brain Interaction—Results From the Rome Foundation Global Epidemiology Study

**DOI:** 10.1002/ueg2.70269

**Published:** 2026-07-29

**Authors:** Jessica Sjölund, Jóhann P. Hreinsson, Brian E. Lacy, Hans Törnblom, Shrikant I. Bangdiwala, Olafur S. Palsson, Ami D. Sperber, Magnus Simren

**Affiliations:** ^1^ Department of Molecular and Clinical Medicine Institute of Medicine Sahlgrenska Academy University of Gothenburg Gothenburg Sweden; ^2^ Department of Medicine Sahlgrenska University Hospital Gothenburg Sweden; ^3^ Division of Gastroenterology and Hepatology Mayo Clinic Jacksonville Florida USA; ^4^ Department of Health Research Methods, Evidence, and Impact McMaster University Hamilton Ontario Canada; ^5^ Center for Functional GI & Motility Disorders University of North Carolina–Chapel Hill Chapel Hill North Carolina USA; ^6^ Faculty of Health Sciences Ben‐Gurion University of the Negev Beer‐Sheva Israel

**Keywords:** abdominal pain, anaemia, constipation, diarrhoea, dyspepsia, dysphagia, haematochezia, irritable bowel syndrome, weight loss

## Abstract

**Background:**

Data on the global prevalence of alarm features in the general population and among individuals with disorders of gut–brain interaction (DGBI) are limited.

**Methods:**

We analysed data from the Rome Foundation Global Epidemiology Study to estimate the prevalence of fourteen self‐reported alarm features in the global population and among individuals with Rome IV defined DGBI, including 54,127 participants from 26 countries. Differences between participants with and without DGBI were evaluated in a sample excluding those with self‐reported organic gastrointestinal disease, using univariable logistic regression. Analyses were stratified by age (< 50 vs. ≥ 50 years).

**Results:**

Alarm features were reported by 63.1% of participants aged < 50 years and 51.8% of those aged ≥ 50 years. Individual self‐reported alarm features ranged from 0.9% to 18.3% in younger participants to 0.3%–13.3% in older participants. Haematemesis was the least frequently reported feature, while a major change in bowel movements was the most frequent in both age groups. Self‐reported alarm features were more frequent among participants with than without DGBI (< 50 years: 75.3% vs. 50.7%; OR 3.0 [2.8–3.1]; ≥ 50 years: 67.5% vs. 38.8%; OR 3.3 [3.1–3.5]). Participants with DGBI were also more likely to report multiple alarm features.

**Discussion:**

Self‐reported alarm features are common in the general population and more prevalent among individuals with DGBI. These findings underscore the importance of interpreting alarm features within a broader clinical context rather than in isolation. Further studies are needed to clarify the diagnostic utility of alarm features in unselected populations, particularly among individuals with DGBI.

## Key Summary

- Summarize the established knowledge on this subject.
  ◦ Alarm features are symptoms, signs, and family history features that may indicate underlying disease and prompt further diagnostic evaluation.
  ◦ Commonly recognised alarm features in gastroenterology include haematemesis, dysphagia, melaena, haematochezia, iron deficiency anaemia, unintentional weight loss, and a family history of gastrointestinal cancer.
  ◦ For disorders of gut–brain interaction, alarm features help guide the need for investigations beyond a positive symptom‐based diagnosis and limited target testing.
  ◦ The prevalence of alarm features in the general population, and among individuals with and without disorders of gut–brain interaction, has not been characterized in large multinational samples.
- What are the significant and/or new findings of this study?
  ◦ Self‐reported alarm features are highly prevalent in the global adult population, with more than half of individuals reporting at least one of the 14 assessed features; reporting is more common among younger individuals.
  ◦ Self‐reported alarm features are more frequently reported among individuals meeting the Rome IV criteria for disorders of gut–brain interaction across all major anatomical segments of the gastrointestinal tract.
  ◦ Individuals meeting the criteria for disorders of gut–brain interaction more often report multiple alarm features.
  ◦ Among individuals with disorders of gut–brain interaction, alarm feature reporting is associated with overlapping disorders, greater somatic symptom burden, greater psychological distress, and increased healthcare utilization.

## 1 Introduction

Alarm features are symptoms, signs, or family history characteristics that may prompt consideration of underlying disease and further diagnostic evaluation, including expedited evaluation when serious underlying disease is suspected [1]. In gastroenterology, commonly recognized alarm features include dysphagia, haematemesis, melaena, haematochezia, iron deficiency anaemia, unintentional weight loss, and a family history of gastrointestinal cancer [1, 2, 3]. Disorders of gut–brain interaction (DGBI) are a group of common gastrointestinal disorders for which clinical guidelines support a positive diagnosis based on symptom criteria (Rome criteria), often with only limited targeted investigations in the absence of alarm features, while recommending further evaluation when alarm features are present [4, 5, 6, 7, 8, 9].

Despite their widespread use in clinical practice, data on the prevalence of alarm features in the general population and among individuals meeting symptom‐based criteria for DGBI remain limited, particularly globally [10, 11, 12, 13]. Understanding how frequently alarm features occur in the general population and among individuals with DGBI may help contextualize their role as clinical markers of concern and inform clinical decision‐making and future diagnostic research.

Using data from the Rome Foundation Global Epidemiology Study (RFGES), we estimated the prevalence of 14 self‐reported alarm features in the global general population and among individuals meeting the Rome IV criteria for DGBI.

## 2 Materials and Methods

### 2.1 Study Population

Data were derived from the RFGES, a cross‐sectional global population‐based study conducted between 2017 and 2018 [4]. The present analyses included all 54,127 adult participants from the internet survey component, representing 26 countries across six continents. A minimum of 2000 participants per country were recruited nationwide by a market research company (Qualtrics LLC) using quota sampling to ensure balanced representation by sex (50% male, 50% female) and age (18–39 years: 40%; 40–64 years: 40%; ≥ 65 years: 20%), with minor deviations from the predefined age quotas in a small number of countries. Detailed methodological information has been published previously [4] and is provided in the Supporting Information S1: Methods. Ethics review of the RFGES was completed for all countries surveyed, and the study was in each case either approved or exempted from ethics oversight due to the anonymity of the subjects. All participants provided electronic informed consent prior to participation in the survey.

### 2.2 Measures

The survey collected self‐reported demographic and anthropometric data. It included the Rome IV Diagnostic Questionnaire (R4DQ) to assess gastrointestinal symptoms [14], the Patient Health Questionnaire‐12 (PHQ‐12) to assess non‐gastrointestinal somatic symptoms [15], and the Patient Health Questionnaire‐4 (PHQ‐4) to screen for anxiety and depression [16]. The survey also included a checklist of self‐reported physician‐diagnosed organic diseases and prior gastrointestinal surgeries, as well as questions on healthcare utilization.

### 2.3 Definitions

#### 2.3.1 Self‐Reported Alarm Features

The self‐reported alarm features assessed were the 14 items appended to the R4DQ to prompt consideration of alternative diagnoses in individuals with symptoms otherwise compatible with DGBI. These alarm features comprise somatic symptoms, signs, and family history features that may indicate gastrointestinal or extra‐intestinal disease beyond DGBI [17]. Psychiatric alarm features were not included. All alarm features and their definitions are presented in Table 1, with corresponding R4DQ items provided in Supporting Information S1: Table S1.

**TABLE 1:** Self‐reported alarm features and their definitions.

Alarm feature	Definition (all based on self‐report)
Neck/throat pain	Persistent and worsening neck or throat pain in the last 3 months
Hoarseness	Persistent or worsening hoarseness of the voice in the last 3 months
Suspected cardiac chest pain	Chest pain on exertion or chest pain related to heart problems two or more times in the last 3 months
Dysphagia	Difficulty swallowing on two or more occasions in the last 3 months
Haematemesis	Vomited blood two or more times in the last 3 months
Black stools	Black stools two or more times in the last 3 months
Haematochezia	Red blood in stools two or more times in the last 3 months
Major change in bowel movements	Major change in bowel movements (change in frequency or consistency) in the last 3 months
Unintentional weight loss	Unintentional weight loss of more than 4.5 kg in the last 3 months
Anaemia/low iron	Physician‐diagnosed low blood count or low iron
Fever	Temperature over 38 degrees centigrade two or more times in the last 3 months
Family history of gastrointestinal cancer	First‐degree relative with a history of cancer of the oesophagus, stomach or colon
Family history of coeliac disease	First‐degree relative with a history of coeliac disease
Family history of inflammatory bowel disease	First degree relative with a history of ulcerative colitis or Crohn's disease

*Note:* Self‐reported alarm features include the 14 items (symptoms, sign, and family history) appended to the Rome IV Diagnostic Questionnaire for adult disorders of gut–brain interaction.

#### 2.3.2 DGBI

Twenty‐two adult DGBI diagnoses were classified according to the Rome IV criteria (Table 2) [18]. ‘Any DGBI’ was defined as meeting the criteria for at least one diagnosis. Twenty diagnoses were categorized into oesophageal, gastroduodenal, bowel, and anorectal disorders. Two diagnoses (centrally mediated abdominal pain syndrome and biliary pain) were not included in analyses by anatomical region due to low prevalence (< 0.1%) [4]. ‘Overlapping DGBI’ was defined as meeting the criteria for diagnoses in at least two anatomical regions (oesophageal, gastroduodenal, bowel, or anorectal). The number of affected regions (range 1–4) was used to characterize the extent of overlap.

**TABLE 2:** Rome IV disorders of gut–brain interaction diagnoses included in the analyses.

Anatomical region	Diagnoses
Oesophageal disorders	Functional chest pain
	Functional heartburn
	Reflux hypersensitivity
	Globus
	Functional dysphagia
Gastroduodenal disorders	Functional dyspepsia
	Belching disorder
	Rumination syndrome
	Chronic nausea syndrome
	Cyclic vomiting syndrome
	Cannabinoid hyperemesis syndrome
Bowel disorders	Irritable bowel syndrome
	Functional constipation
	Opioid‐induced constipation
	Functional diarrhoea
	Functional bloating/distention
	Unspecified functional bowel disorder
Centrally mediated disorders of gastrointestinal pain	Centrally mediated abdominal pain syndrome
Gallbladder and sphincter of Oddi disorders	Functional biliary pain
Anorectal disorders	Faecal incontinence
	Levator ani syndrome
	Proctalgia fugax

#### 2.3.3 Geographical Regions

For regional analyses, countries were grouped into six geographical regions according to previous RFGES publications (Supporting Information S1: Method) [4]. Australia and South Africa were analysed separately, as they did not align with the predefined regional classifications.

#### 2.3.4 Somatic Symptoms, Psychological Factors, and Healthcare Utilization

Non‐gastrointestinal somatic symptom burden was assessed as the PHQ‐12 score, excluding the item on menstrual problems in women. Psychological distress was assessed using the PHQ‐4 score. Healthcare utilization was assessed as the frequency of doctor visits using the item: ‘How often do you go to a doctor for your health?’.

### 2.4 Data Analysis

We calculated the prevalence of any self‐reported alarm feature, the number of alarm features reported, and the prevalence of individual alarm features in the overall study population and among participants with any DGBI compared with those without DGBI. We also assessed the prevalence of selected individual alarm features among participants with oesophageal, gastroduodenal, bowel, and anorectal DGBI compared with those without DGBI, restricting analyses to alarm features considered clinically relevant to each DGBI group (Supporting Information S1: Table S2). Analyses were stratified by age (< 50 vs. ≥ 50 years), as age is a key factor in the clinical evaluation of alarm features [1].

For analyses involving DGBI, participants with self‐reported organic or structural gastrointestinal disease (e.g., ulcerative colitis, Crohn's disease, coeliac disease, gastrointestinal cancer, peptic ulcer disease, diverticulitis, and prior bowel resection) were excluded from the analytic sample (*n* = 4094) to reduce potential confounding by conditions that may independently be associated with alarm features.

As a post hoc sensitivity analysis, we repeated the analyses of any and multiple alarm features using a restricted set of traditional gastrointestinal alarm features comprising dysphagia, haematemesis, black stools, haematochezia, anaemia/low iron, unintentional weight loss, and family history of gastrointestinal cancer.

Exploratory analyses among participants with any DGBI assessed factors associated with alarm feature reporting.

### 2.5 Statistical Analysis

Categorical variables are presented as proportions (%) with 95% confidence intervals (CI), and continuous variables as means with 95% CI. Differences in the prevalence of self‐reported alarm features between participants with and without DGBI were assessed using univariable logistic regression, with odds ratios (OR) and 95% CI reported as effect measures of between‐group differences. Associations between independent factors and alarm feature reporting among participants with any DGBI were assessed using univariable and multivariable logistic regression, presented as OR with 95% CI. Analyses were performed using R (R Foundation for Statistical Computing, Vienna, Austria), version 4.5.2.

## 3 Results

### 3.1 Self‐Reported Alarm Features in the General Population

#### 3.1.1 Prevalence

Among the 54,127 participants, 63.0% of those aged < 50 years and 51.7% of those aged ≥ 50 years reported at least one of 14 assessed self‐reported alarm features. Approximately one‐third of the younger and one‐quarter of the older participants reported more than one alarm feature (Table 3). Restricting analyses to a set of seven traditional gastrointestinal alarm features reduced prevalence estimates, with 43.2% of participants aged < 50 years and 34.4% of those aged ≥ 50 years reporting at least one feature, and 13.2% and 8.6%, respectively, reporting two or more features (Supporting Information S1: Table S3).

**TABLE 3:** Prevalence and number of self‐reported alarm features in the global population, by age group.

Number of self‐reported alarm features	Age < 50 years (*n* = 34,027)	Age ≥ 50 years (*n* = 20,100)
Any, % (95% CI)	63.0 (62.5–63.5)	51.7 (51.0–52.3)
1, % (95% CI)	30.2 (29.7–30.7)	28.4 (27.8–29.1)
≥ 2, % (95% CI)	32.7 (32.2–33.2)	23.1 (22.6–23.7)
≥ 3, % (95% CI)	14.8 (14.4–15.2)	8.9 (8.5–9.3)
Mean (95% CI)	1.2 (1.2–1.3)	0.9 (0.9–0.9)

*Note:* Self‐reported alarm features include the 14 items (symptoms, signs, and family history) appended to in the Rome IV Diagnostic Questionnaire for adult disorders of gut–brain interaction.

Abbreviations: CI, confidence interval; *n*, number.

**FIGURE 1:**
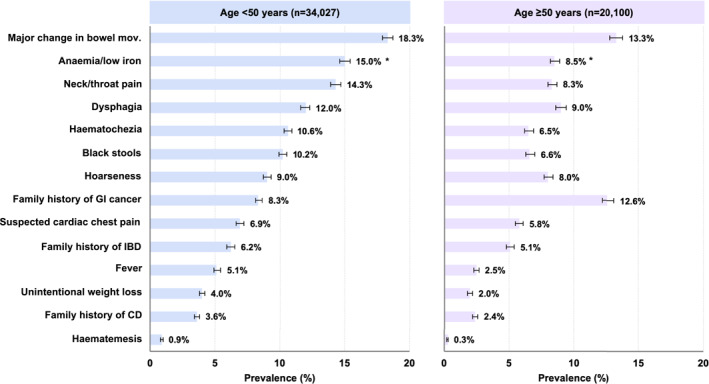
Prevalence of individual self‐reported alarm features in the global population by age group. Self‐reported alarm features include the 14 items (symptoms, signs, and family history) appended to the rome IV diagnostic questionnaire for adult disorders of gut–brain interaction. Error bars represent 95% confidence intervals. * Sex‐stratified prevalence: < 50 years, female 22.1%, male 7.4%; ≥ 50 years, female 11.2%, male 6.4%.CD, coeliac disease; GI, gastrointestinal; IBD, inflammatory bowel disease; mov, movements; n, number.

The prevalence of individual self‐reported alarm features ranged from 0.9% to 18.3% in participants aged < 50 years and from 0.3% to 13.3% in those aged ≥ 50 years (Figure 1, Supporting Information S1: Table S4). Major change in bowel movements was the most commonly reported alarm feature in both age groups, whereas haematemesis was the least commonly reported. Most individual alarm features were reported with similar or higher frequency in participants aged < 50 years, except for a family history of gastrointestinal cancer, which was more commonly reported among those aged ≥ 50 years.

#### 3.1.2 Regional Variation

The prevalence of any self‐reported alarm feature varied across geographical regions. Among participants aged < 50 years, it ranged from 51.8% in Australia to 71.6% in Latin America, and among those aged ≥ 50 years from 41.1% in Australia to 64.6% in Eastern Europe (Supporting Information S1: Table S5). Most individual alarm features also varied across regions (Supporting Information S1: Table S5).

#### 3.1.3 Participant Characteristics

Participants reporting at least one self‐reported alarm feature were slightly younger (mean age 43 vs. 47 years) than those reporting none and included a slightly lower proportion of men (49.2% vs. 53.4%). Mean body mass index and years of education were similar between the groups (Supporting Information S1: Table S6).

### 3.2 Self‐Reported Alarm Features in DGBI

#### 3.2.1 Any DGBI

Among 50,033 participants without organic or structural gastrointestinal disease (92.4% of the total study population), the prevalence of any self‐reported alarm feature was higher among participants with DGBI than among those without DGBI across both age groups (Table 4). Participants with DGBI also more frequently reported multiple alarm features (Table 4). This pattern was consistent across all individual alarm features (Supporting Information S1: Table S7) and geographical regions (Supporting Information S1: Table S8).

**TABLE 4:** Prevalence and number of self‐reported alarm features among individuals with and without DGBI, by age group.

	Age < 50 years			Age ≥ 50 years		
Number of self‐reported alarm features	Any DGBI (*n* = 14,174)	No DGBI (*n* = 17,995)	OR (95% CI)	Any DGBI (*n* = 6494)	No DGBI (*n* = 11,370)	OR (95% CI)
Any, % (95% CI)	75.2 (74.5–75.9)	50.7 (49.9–51.4)	**3.0 (2.8–3.1)**	67.5 (66.3–68.6)	38.7 (37.8–39.6)	**3.3 (3.1–3.5)**
1, % (95% CI)	30.8 (30.0–31.6)	30.9 (30.2–31.6)	1.0 (0.9–1.0)	32.7 (31.6–33.9)	26.2 (25.4–27.0)	**1.4 (1.3–1.5)**
≥ 2, % (95% CI)	44.4 (43.6–45.3)	19.8 (19.2–20.3)	**3.2 (3.1–3.4)**	34.7 (33.6–35.9)	12.5 (11.9–13.1)	**3.7 (3.4–4.0)**
≥ 3, % (95% CI)	22.5 (21.9–23.2)	5.8 (5.5–6.2)	**4.7 (4.4–5.1)**	14.9 (14.1–15.8)	2.9 (2.6–3.2)	**5.9 (5.2–6.7)**
Mean (95% CI)	1.6 (1.6–1.7)	0.8 (0.8–0.8)	N/A	1.3 (1.3–1.3)	0.6 (0.5–0.6)	N/A

*Note:* OR are unadjusted and should be interpreted as descriptive measures of between‐group differences. OR values with 95% CI not including 1.0 are shown in bold. Participants with a reported history of organic or structural gastrointestinal disease were excluded from the analyses. Self‐reported alarm features include the 14 items (symptoms, signs, and family history) appended to the Rome IV Diagnostic Questionnaire for adult DGBI.

Abbreviations: CI, confidence interval; DGBI, disorder of gut–brain interaction; *n*, number; OR, odds ratio.

Restricting analyses to a set of seven traditional gastrointestinal alarm features yielded lower prevalence estimates, but the overall pattern remained unchanged, with participants with DGBI more likely to report both any and multiple alarm features than those without DGBI (Supporting Information S1: Table S9).

Among excluded participants with organic or structural gastrointestinal disease, additionally excluding those with concurrent DGBI, the prevalence of any self‐reported alarm feature was 88.4% among participants aged < 50 years and 67.5% among those aged ≥ 50 years (Supporting Information S1: Table S10).

#### 3.2.2 Anatomical DGBI Regions

**FIGURE 2:**
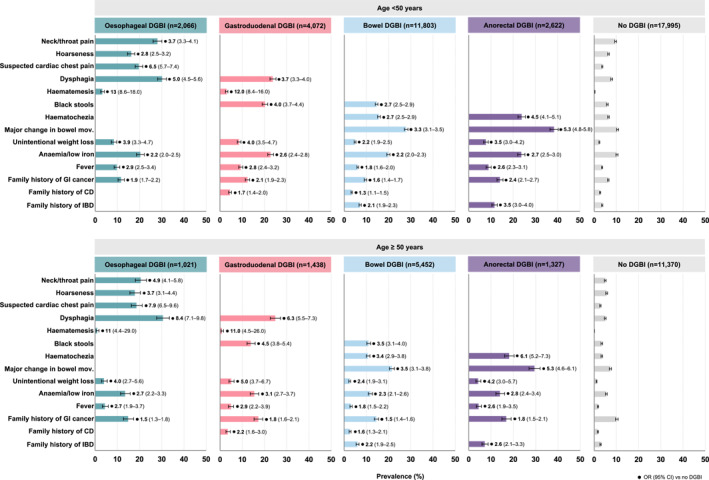
Prevalence of selected individual self‐reported alarm features in participants with and without DGBI by anatomical region and age group. Participants with a reported history of organic or structural gastrointestinal disease were excluded from the analyses. Self‐reported alarm features include the 14 items (symptoms, signs, and family history) appended to the rome IV diagnostic questionnaire for adult DGBI. Only self‐reported alarm features considered clinically relevant to the diagnoses included in each anatomical region are shown. Error bars represent 95% confidence intervals. OR are unadjusted and should be interpreted as descriptive measures of between‐group differences. OR values with 95% CI not including 1.0 are shown in bold. CD, coeliac disease; GI, gastrointestinal; IBD, inflammatory bowel disease; mov, movements; n, number.

Across all anatomical groups, all selected individual alarm features were more frequently reported by participants with DGBI than by those without DGBI in both age groups (Figure 2). The magnitude of these differences varied across alarm features. In oesophageal and gastroduodenal DGBI, the largest differences were observed for haematemesis, although the absolute prevalence was low in both groups. In bowel and anorectal DGBI, major change in bowel movements and haematochezia showed the largest differences and were also among the most frequently reported alarm features. In addition, black stools showed one of the larger differences in bowel DGBI.

#### 3.2.3 Associated Factors

In exploratory analyses among participants meeting criteria for DGBI, overlapping DGBI was strongly associated with reporting of any alarm feature across both age groups, with increasing odds observed across the number of affected anatomical regions (Table 5). Higher psychological distress, greater non‐gastrointestinal somatic symptom burden, and more frequent doctor visits were also associated with alarm feature reporting.

**TABLE 5:** Factors associated with the self‐report of any alarm feature among individuals with disorders of gut–brain interaction, by age group.

Factor	Age < 50 years				Age ≥ 50 years			
	No alarm features (*n* = 3502)	Any alarm feature (*n* = 10,656)	OR (95% CI)	aOR^a^ (95% CI)	No alarm feature (*n* = 2105)	Any alarm feature (*n* = 4381)	OR (95% CI)	aOR^a^ (95% CI)
Sex, % (95% CI)								
Female	60.7 (59.1–62.3)	58.4 (57.5–59.3)	Ref	Ref	54.6 (52.4–56.7)	52.4 (50.9–53.9)	Ref	Ref
Male	39.3 (37.7–40.9)	41.6 (40.7–42.5)	**1.1 (1.0–1.2)**	**1.3 (1.2–1.4)**	45.4 (43.3–47.6)	47.6 (46.1–49.1)	**1.1 (1.0–1.2)**	**1.3 (1.2–1.5)**
Number of affected anatomical DGBI regions, % (95% CI)								
1 region	82.8 (81.5–84.1)	62.7 (61.8–63.6)	Ref	Ref	80.5 (78.8–82.2)	63.1 (61.7–64.5)	Ref	Ref
2 regions	14.0 (12.9–15.3)	24.6 (23.8–25.5)	**2.3 (2.1–2.6)**	**1.8 (1.6–2.0)**	16.5 (15.0–18.2)	25.1 (23.8–26.4)	**1.9 (1.7–2.2)**	**1.5 (1.3–1.7)**
3 regions	2.7 (2.2–3.4)	9.3 (8.8–9.9)	**4.5 (3.6–5.6)**	**2.8 (2.3–3.5)**	2.5 (1.9–3.3)	9.0 (8.2–9.9)	**4.6 (3.4–6.2)**	**3.1 (2.3–4.2)**
4 regions	0.4 (0.2–0.7)	3.3 (3.0, 3.7)	**11.8 (6.8–20.5)**	**5.6 (3.3–10.3)**	0.5 (0.2–0.9)	2.9 (2.4–3.4)	**7.7 (4.0–14.6)**	**3.8 (2.0–7.8)**
Psychological distress, mean PHQ‐4 score (95% CI)	3.3 (3.2–3.4)	4.5 (4.4–4.5)	**1.1 (1.1–1.1)**	**1.0 (1.0–1.1)**	2.2 (2.1–2.3)	3.3 (3.2–3.4)	**1.2 (1.1–1.2)**	**1.1 (1.0–1.1)**
Non‐GI somatic symptom severity, mean PHQ‐12 score (95% CI)	5.2 (5.1–5.3)	7.2 (7.2–7.3)	**1.2 (1.2–1.2)**	**1.1 (1.1–1.2)**	4.8 (4.6–4.9)	6.7 (6.5–6.8)	**1.2 (1.2–1.2)**	**1.1 (1.1–1.2)**
Number of doctor visits, % (95% CI)								
Never	4.5 (3.8–5.2)	3.1 (2.8–3.5)	Ref	Ref	2.1 (1.6–2.9)	1.4 (1.1–1.8)	Ref	Ref
Less than once per year	22.4 (21.0–23.8)	15.8 (15.2–16.6)	1.0 (0.8–1.2)	1.0 (0.8–1.3)	14.3 (12.8–15.8)	11.4 (10.5–12.4)	1.2 (0.8–1.9)	1.2 (0.8–1.9)
Once per year	17.0 (15.7–18.3)	13.4 (12.8–14.1)	1.1 (0.9–1.4)	1.2 (0.9–1.5)	16.1 (14.5–17.7)	11.0 (10.1–12.0)	1.0 (0.7–1.6)	1.1 (0.7–1.6)
A few times per year	47.7 (46.0–49.3)	53.1 (52.2–54.1)	**1.6 (1.3–1.9)**	**1.5 (1.2–1.8)**	54.6 (52.4–56.7)	53.8 (52.4–55.3)	**1.5 (1.0–2.2)**	1.3 (0.9–2.0)
Once a month	8.5 (7.6–9.5)	14.5 (13.8–15.1)	**2.4 (1.9–3.0)**	**1.6 (1.3–2.1)**	13.0 (11.6–14.5)	22.4 (21.1–23.6)	**2.6 (1.8–4.0)**	**1.8 (1.2–2.8)**

*Note:* Participants with a reported history of organic or structural gastrointestinal disease were excluded from the analyses. Self‐reported alarm features include the 14 items (symptoms, signs, and family history) appended to the Rome IV Diagnostic Questionnaire for adult DGBI. OR values with 95% CI not including 1.0 are shown in bold. Sensitivity analyses excluding participants reporting anaemia/low iron as their sole alarm feature (self‐reported physician diagnosis) slightly attenuated the association between health‐care utilization and the self‐report of any alarm feature, but did not remove it (data not shown).

Abbreviations: aOR, adjusted odds ratio. CI, confidence interval; DGBI, disorder of gut–brain interaction; *n*, number; OR, odds ratio.

^a^ Adjusted for the other factors in the table.

## 4 Discussion

In this large multinational population‐based study, self‐reported alarm features were common, with more than half of individuals reporting at least one of the 14 assessed features and a substantial proportion reporting multiple features. Restricting analyses to a set of more traditional gastrointestinal alarm features resulted in lower prevalence estimates but still identified at least one reported alarm feature in approximately two‐fifths of younger and one‐third of older participants. The prevalence of individual self‐reported alarm features varied widely and was generally higher among younger individuals. All self‐reported alarm features were more frequently reported among individuals meeting the Rome IV criteria for DGBI than among those not meeting these criteria, and individuals with DGBI also reported a higher burden of multiple alarm features.

Few studies have assessed a broad range of self‐reported alarm features in community samples [10, 11, 12, 13], and none in a large multinational sample using standardised methodology across countries. Direct comparison across studies is challenging because prevalence estimates likely depend on the alarm features assessed, their definitions, recall periods, study populations, and geographical and cultural settings. Nevertheless, our findings that self‐reported features are commonly reported in the population are broadly consistent with previous population‐based studies [10, 11, 12, 13]. For example, a UK study reported that more than half of individuals aged ≥ 50 years experienced at least one of 21 symptoms possibly indicative of cancer over a 1‐year period, including features suggestive of upper gastrointestinal cancer (29%) and colorectal cancer (17%) [10]. Danish studies, using shorter recall periods and a slightly broader age range, similarly reported a high prevalence of colorectal cancer‐related alarm features (up to 39%) alongside lower occurrence of upper gastrointestinal alarm features (up to 8%) [11, 12].

The prevalence of individual self‐reported alarm features varied substantially, ranging from < 1%–18% for gastrointestinal symptoms, 6%–14% for non‐gastrointestinal symptoms, 2%–15% for non‐specific symptoms and signs, and 2%–13% for a family history of gastrointestinal disease. Although some self‐reported alarm features, such as haematemesis, were rare, several others traditionally considered clinically concerning–including dysphagia, haematochezia, anaemia/low iron, and black stools–were reported by up to one in ten participants. The prevalence of reported haematemesis [10, 13] and fever [13] was broadly consistent with previous studies, whereas the prevalence of reported dysphagia [10, 13, 19, 20], major change in bowel movements [10, 13, 21], haematochezia [10, 13, 21, 22, 23], and unintentional weight loss [10, 13, 22, 24] fell within the broad range reported in the literature. Of note, although ‘major change in bowel movements’ is included as an alarm feature in NICE guidelines [1], its role as an alarm feature remains debated [25]. The prevalence of self‐reported black stools appeared higher than in most previous reports [12, 13, 23], which may reflect differences in definitions and recall periods.

Most individual alarm features were more frequently reported among individuals aged < 50 years compared with those aged ≥ 50 years, except for a family history of gastrointestinal cancer. This pattern is consistent with other population‐based studies [11, 12], but contrasts with findings from clinical settings [26], where alarm features are more commonly reported among older individuals. This difference may reflect age‐related variation in healthcare‐seeking behaviour, referral patterns, and survival bias [10, 11, 12, 27].

Among individuals meeting the criteria for DGBI, reporting of any alarm feature was more common than among those not meeting the criteria across age groups and geographical regions, and individuals with DGBI also more often reported multiple alarm features. Part of this difference may reflect overlap between certain alarm features and DGBI symptom profiles, such as dysphagia in oesophageal disorders or major change in bowel movements in bowel disorders [28, 29]. However, all assessed alarm features were more frequently reported among individuals with DGBI, including features not typically considered part of the DGBI symptom spectrum, such as those related to gastrointestinal bleeding.

These findings are consistent with previous studies reporting a high prevalence of alarm features in IBS, with estimates ranging from 70% to 84% [30, 31, 32, 33]. Where comparison of individual alarm features was possible, prevalence estimates in our study were generally lower than those reported in IBS [30, 31, 32, 33, 34, 35] and functional dyspepsia [34] populations from secondary and tertiary care settings, possibly reflecting referral bias. As most previous studies were conducted in referral settings and focused primarily on IBS, our findings extend the literature by demonstrating increased alarm feature reporting across all major anatomical DGBI groups in a large multinational population‐based sample.

Several factors may contribute to the increased prevalence of self‐reported alarm features among individuals meeting DGBI criteria. One possibility is undiagnosed underlying organic disease. However, studies in IBS populations suggest that although alarm features may increase the likelihood of organic disease in certain subgroups [31], most patients ultimately diagnosed with IBS also report alarm features [30, 32, 33], indicating that this is unlikely to fully explain the observed pattern. Consistent with this, a study of patients fulfilling Rome IV criteria for functional bowel disorders identified clinically relevant colonoscopy findings in only a minority, despite alarm features being present in almost all patients [36]. Alternatively, the higher prevalence of alarm feature reporting may reflect differences in symptom perception, reporting, or overall symptom burden. In our exploratory analyses, alarm feature reporting was strongly associated with overlapping DGBI, with increasing odds across the number of affected anatomical regions. Alarm feature reporting was also associated with greater psychological distress, non‐gastrointestinal somatic symptom severity, and health‐care utilization, although the effect sizes for psychological distress and somatic symptom severity were small. Taken together, these findings suggest that alarm feature reporting may partly reflect a broader pattern of symptom burden and reporting, in addition to the possibility of underlying organic disease.

The concept of identifying alarm features is to facilitate early detection of potentially serious underlying diseases and prompt expedited diagnostic evaluation. Although the present study does not allow assessment of the predictive value or clinical relevance of the alarm features assessed, several alarm features were commonly reported in the general population. In the context of previous studies demonstrating variable diagnostic accuracy of individual alarm features [21, 24, 25, 37, 38, 39], these findings underscore the importance of interpreting alarm features within a broader clinical context rather than in isolation. This may be particularly relevant in DGBI, where studies have shown that incorporating alarm features into diagnostic criteria for disorders such as IBS may increase specificity but at the cost of substantially reduced sensitivity [32, 33, 40], supporting current criteria that do not require their absence [3]. However, as clinical guidelines continue to recommend assessment of alarm features and further evaluation when present [6, 7, 8, 9], the high prevalence of self‐reported alarm features observed in our study suggests that such features may frequently be encountered among individuals meeting criteria for DGBI, potentially creating challenges for the implementation of a positive diagnostic approach. Further research is needed to clarify the diagnostic utility of individual and combined alarm features, particularly in unselected DGBI populations, to better inform diagnostic investigations.

Strengths of this study include the large sample size, the multinational population‐based design, and the use of standardized methodology across participating countries. The internet‐based survey, with built‐in quality control measures and quota sampling, ensured a demographically balanced sample and high‐quality data.

Several limitations should be acknowledged. First, alarm features were assessed using self‐reported questionnaire items not validated for this purpose. Some questionnaire items may represent broader constructs than those typically considered clinically relevant alarm features (e.g., fever, black stools, anaemia/low iron), and reported alarm features may not correspond to those considered clinically relevant following medical consultation, where symptom characteristics, clinical context, and alternative explanations can be explored in greater detail. We were also unable to assess their predictive value for underlying organic disease. Second, although DGBI were classified according to the Rome IV criteria using the R4DQ, classification was not clinically verified. While participants with known organic or structural gastrointestinal disease were excluded, misclassification due to undiagnosed conditions cannot be excluded. Third, the cross‐sectional design precludes conclusions regarding directionality or causality. Finally, selection bias cannot be excluded. Although efforts were made to minimize the selection of individuals with greater gastrointestinal symptom burden by describing the survey as a general health survey, symptomatic individuals may still have been more likely to participate [41], potentially overestimating the prevalence of self‐reported alarm features. Information on invited non‐responders was unavailable.

## 5 Conclusions

Self‐reported alarm features are common in the general population and even more so among individuals meeting the Rome IV criteria for DGBI. These findings underscore the need to interpret alarm features within a broader clinical context rather than in isolation. Further research is needed to clarify the diagnostic utility of alarm features in unselected populations, particularly among individuals with DGBI, to better inform clinical decision‐making and guideline development.

## Conflicts of Interest

These authors disclose the following: Brian E Lacy has received research grants from Ironwood, has served as an advisory board member for GSK and Ferring, and is a member of the Rome Foundation Board of Trustees. Hans Törnblom has received consulting fees for Medifactia AB, PRO.MED.CS, Purpose Pharma, and Vipun Medical, payment/honoraria for lectures from Galapagos Biopharma and Mylan AB, and has served as an advisory board member for Cinclus Pharma and Dr. Falk Pharma GmbH. Magnus Simrén has received research grants from BioGaia, consulting fees from Biocodex, Tillotts, BioGaia, Renapharma, and Alfasigma, payment/honoraria for lectures from Tillotts, Takeda, Biocodex, Sanofi, AbbVie, Janssen Immunology, Pfizer, BioGaia, Renapharma, Mayoly, and Bromatech, has served as an advisory board member for Biocodex, Tillotts, BioGaia, Renapharma, and Alfasigma, and is a member of the Rome Foundation Board of Directors. The remaining authors disclose no conflicts.

## Supporting information


Supporting Information S1

## Data Availability

The data that support the findings of this study are available from the corresponding author upon reasonable request.
