# Supplementary material for: Global Prevalence of Self‐Reported Alarm Features in the General Population and Among Individuals With Disorders of Gut–Brain Interaction—Results From the Rome Foundation Global Epidemiology Study
Source: United European Gastroenterol J. 2026 Jul 29;14(7):e70269. doi: 10.1002/ueg2.70269 (PMC13417726; doi:10.1002/ueg2.70269)
Supplement: Supplementary file 1 — Supporting Information S1 [file UEG2-14-e70269-s001.docx]

**Supplement**

Global Prevalence of Self-Reported Alarm Features in the General Population and Among Individuals with Disorders of Gut-Brain Interaction— Results from the Rome Foundation Global Epidemiology Study

**Supplementary Methods**

- 1. **Study design and setting**
  2. **Participants and recruitment**
  3. **Survey administration and data quality assurance**
  4. **Questionnaire translation**
  5. **Definition of geographical regions**

**Supplementary Tables**

- **Table 1:** Rome IV Diagnostic Questionnaire items used to assess self-reported alarm features
- **Table 2:** Self-reported alarm features considered clinically relevant for each DGBI group
- **Table 3.** Prevalence and number of self-reported traditional gastrointestinal alarm features in the global population, by age group
- **Table 4:** Prevalence of any and individual self-reported alarm features in the global population (not age-stratified)
- **Table 5:** Regional prevalence of any and individual self-reported alarm features by age group
- **Table 6:** Characteristics of participants with and without any self-reported alarm feature
- **Table 7:** **Prevalence of individual self-reported alarm features among individuals with and without DGBI, by age group**
- **Table 8:** Regional prevalence of any self-reported alarm feature by DGBI status and age group
- **Table 9.** Prevalence and number of self-reported traditional gastrointestinal alarm features among individuals with and without DGBI, by age group
- **Table 10: Prevalence of any and individual self-reported alarm features in individuals with self-reported history of organic gastrointestinal disease without concurrent DGBI**

1. **SUPPLEMENTARY METHODS**

**1.1 Study design and setting**

The Rome Foundation Global Epidemiology Study (RFGES) was designed to determine the global prevalence of disorders of gut-brain interaction (DGBI) and examine associated factors. The study was conducted in 33 countries across the globe–with some regional underrepresentation in Africa and the Middle East. Data were collected either via internet survey only (n=24), household survey (n=7) only, or both methods (n=2), depending on country-specific internet-access. The present study used data from the internet survey component of the Rome Foundation Global Epidemiology Study, including 54,127 adult participants from 26 countries (Canda, United states, Argentina, Brazil, Colombia, Mexico, Belgium, France, Germany, The Netherlands, Italy, Spain, Sweden, United Kingdom, Poland, Romania, Russia, Egypt, Israel, Turkey, China, Japan, Korea, Singapore, South Africa, and Australia).

**1.2 Participants and recruitment**

Participants were recruited from pre-existing online panels of individuals registered to complete surveys and were invited via email through a global market research company (Qualtrics, LLC; Provo, UT, USA). To minimize selection bias related to gastrointestinal symptoms, the survey was described as a general health survey without reference to gastrointestinal conditions. Participants provided electronic informed consent and completed the survey anonymously. Compensation was provided in the form of points redeemable for rewards according to Qualtrics policies.

**1.3 Survey administration and data quality assurance**

Quota sampling was used to ensure balances and age within each country, as described in the main Methods. Several measures were implemented to ensure data quality, including two attention check questions, two duplicate gastrointestinal symptom items to identify inconsistent responses, and automated monitoring of unusually rapid survey completion. Participants failing any of these checks were automatically excluded. The survey platform enforced completion of mandatory items and applied automated skip patterns. Electronic cookies and unique panel identifiers were used to minimize duplicate participation.

**1.4 Questionnaire translation**

The questionnaire underwent a translatability assessment and was translated into 21 languages with cognitive validation by a professional translation company (TransPerfect, Inc.; New York, NY, USA). Country principal investigators reviewed translations for linguistic and cultural appropriateness, and adaptions were made where necessary to account for regional language variation.

**1.5 Definition of geographical regions**

Countries were grouped into the following geographical regions according to previous RFGES publications: North America (Canada and the United States), Latin America (Argentina, Brazil, Colombia, and Mexico), Western Europe (Belgium, France, Germany, The Netherlands, Italy, Spain, Sweden, and the United Kingdom), Eastern Europe (Poland, Romania, and Russia), the Middle East (Egypt, Israel, and Turkey), and Asia (China, Japan, Korea, and Singapore).

| **Supplementary Table 1. Questionnaire items used to assess self-reported alarm features (appended to the Rome IV Diagnostic Questionnaire)** | |
| --- | --- |
| **Alarm feature** | **Questionnaire item** |
| Neck/throat pain | In the last three months, have you had any of the following symptoms: Persistent and worsening neck or throat pain? |
| Hoarseness | In the last three months, have you had any of the following symptoms: Persistent or worsening hoarseness of the voice? |
| Suspected cardiac chest pain | In the last three months, have you had any of the following symptoms: Chest pain on exertion or chest pain related to heart problems two or more times? |
| Dysphagia | In the last three months, have you had any of the following symptoms: Difficulty swallowing on two or more occasions? |
| Haematemesis | In the last three months, have you had any of the following symptoms: Vomited blood two or more times? |
| Black stools | In the last three months, have you had any of the following symptoms: Black stools two or more times? |
| Haematochezia | In the last three months, have you had any of the following symptoms: Red blood in your stools two or more times? |
| Major change in bowel movements | In the last three months, have you had any of the following symptoms: Major change in bowel movements (change in frequency or consistency)? |
| Unintentional weight loss | In the last three months, have you had any of the following symptoms: Unintentional weight loss of more than 10 pounds (4.5 kilograms)? |
| Anaemia/low iron | Have you been told by your doctor that you are anaemic (have low blood count or low iron)? |
| Fever | In the last three months, have you had any of the following symptoms: Temperature over 99 degrees Fahrenheit (38 degrees centigrade) two or more times? |
| Family history of gastrointestinal cancer | Do you have a parent, brother or sister who has (or had) one or more of the following: cancer of the oesophagus, stomach or colon. |
| Family history of coeliac disease | Do you have a parent, brother or sister who has (or had) one or more of the following: Coeliac disease |
| Family history of inflammatory bowel disease | Do you have a parent, brother or sister who has (or had) one or more of the following: Ulcerative colitis or Crohn’s disease. |

| **Supplementary Table 2. Self-reported alarm features considered clinically relevant for each anatomical disorder of gut-brain interaction group** | |
| --- | --- |
| **Anatomical region** | **Self-reported alarm features** |
| **Oesophageal disorders** | Neck/throat pain  Hoarseness  Suspected Cardiac chest pain  Haematemesis  Unintentional weight loss  Anaemia/low iron  Fever  Family history of gastrointestinal cancer |
| **Gastroduodenal disorders** | Dysphagia  Haematemesis  Black stools  Unintentional weight loss  Anaemia/low iron  Fever  Family history of gastrointestinal cancer  Family history of coeliac disease |
| **Bowel disorders** | Black stools  Haematochezia  Major change in bowel movements  Unintentional weight loss  Anaemia/low iron  Fever  Family history of gastrointestinal cancer  Family history of coeliac disease  Family history of inflammatory bowel disease |
| **Anorectal disorders** | Haematochezia  Major change in bowel movements  Unintentional weight loss  Anaemia/low iron  Fever  Family history of gastrointestinal cancer  Family history of inflammatory bowel disease |

| **Supplementary Table 3. Prevalence and number of self-reported traditional gastrointestinal alarm features in the global population, by age group** | | |
| --- | --- | --- |
| **Number of self-reported alarm features** | **Age <50 years**  **(n=34,027)** | **Age ≥50 years**  **(n=20,100)** |
| Any, % (95% CI) | 43.2  (42.7–43.8) | 34.4  (33.7–35.1) |
| **1,** % (95% CI) | 30.0  (29.5–30.5) | 25.8  (25.2–26.4) |
| **≥2,** % (95% CI) | 13.2  (12.9–13.6) | 8.6  (8.2–9.0) |
| **≥3,** % (95% CI) | 3.3  (3.2–3.5) | 2.0  (1.8–2.2) |
| Mean (95% CI) | 0.6  (0.6–0.6) | 0.5  (0.4–0.5) |
| Self-reported alarm features include the items on dysphagia, haematemesis, black stools, haematochezia, anaemia/low iron, unintentional weight loss, and family history of gastrointestinal cancer appended to in the Rome IV Diagnostic Questionnaire for adult disorders of gut-brain interaction. *Abbreviations:* CI, confidence interval; n, number. | | |

| **Supplementary Table 4. Prevalence of any and individual self-reported alarm features in the global population (not age-stratified)** | |
| --- | --- |
| **Self-reported alarm features** | **N=54,127**  **% (95% CI)** |
| Any | 58.9  (58.5–59.3) |
| Neck/throat pain | 12.1  (11.8–12.4) |
| Hoarseness | 8.6  (8.4–8.9) |
| Suspected cardiac chest pain | 6.5  (6.3–6.7) |
| Dysphagia | 10.9  (10.6–11.1) |
| Haematemesis | 0.6  (0.6–0.7) |
| Black stools | 8.8  (8.6–9.1) |
| Haematochezia | 9.1  (8.8–9.3) |
| Major change in bowel movements | 16.5  (16.1–16.8) |
| Unintentional weight loss | 3.2  (3.1–3.4) |
| Anaemia/low iron | 12.6  (12.3–12.9) |
| Fever | 4.1  (4.0–4.3) |
| Family history of gastrointestinal cancer | 9.9  (9.7–10.2) |
| Family history of coeliac disease | 3.1  (3.0–3.3) |
| Family history of inflammatory bowel disease | 5.8  (5.6–6.0) |
| *Abbreviations:* CI, confidence interval; n, number. | |

| **Supplementary Table 5. Regional prevalence of any and individual self-reported alarm features by age group (heat map representation)** | | | | | | | | | | | | | | | | |
| --- | --- | --- | --- | --- | --- | --- | --- | --- | --- | --- | --- | --- | --- | --- | --- | --- |
| **Self-reported alarm features** | **Asia** | | **Eastern Europe** | | **Latin America** | | **Middle East** | | **North America** | | **Western Europe** | | **Australia** | | **South Africa** | |
|  | **% (95% CI)** | | **% (95% CI)** | | **% (95% CI)** | | **% (95% CI)** | | **% (95% CI)** | | **% (95% CI)** | | **% (95% CI)** | | **% (95% CI)** | |
|  | **Age <50y** | **Age ≥50y** | **Age <50y** | **Age ≥50y** | **Age <50y** | **Age ≥50y** | **Age <50y** | **Age ≥50y** | **Age <50y** | **Age ≥50y** | **Age <50y** | **Age ≥50y** | **Age <50y** | **Age ≥50y** | **Age <50y** | **Age ≥50y** |
|  | **n=6,507** | **n=2,980** | **n=3,880** | **n=2,226** | **n=5,059** | **n=3,010** | **n=4,785** | **n=1,257** | **n=2,087** | **n=1,965** | **n=9,104** | **n=7,210** | **n=9,104** | **n=7,210** | **n=9,104** | **n=7,210** |
| **Any** | 58.2 (57.0– 59.4) | 50.9 (49.1–52.7) | 68.0 (66.5–69.4) | 64.6 (62.6–66.6) | 71.6 (70.3–72.9) | 61.7 (60.0–63.5) | 71.2 (69.9–72.5) | 58.0 (55.2–60.7) | 57.5 (55.3–59.6) | 46.7 (44.5–49.0) | 57.2 (56.2–58.2) | 45.2 (44.0–46.3) | 51.8 (48.8–54.8) | 41.1 (38.0–44.3) | 68.1 (65.6–70.4) | 58.7 (54.2–63.1) |
| **Neck/throat pain** | 10.5 (9.8–11.3) | 7.1 (6.3–8.1) | 18.0  (16.8–19.3) | 13.5 (12.1–15.0) | 20.8 (19.7–21.9) | 12.9 (11.7–14.1) | 15.1 (14.1–16.1) | 8.4 (7.0–10.1) | 7.8 (6.7–9.1) | 4.0 (3.2–5.0) | 13.6 (12.9–14.3) | 7.1 (6.5–7.7) | 8.8 (7.2–10.7) | 4.6 (3.4–6.2) | 14.3 (12.6–16.1) | 7.3 (5.2–10.1) |
| **Hoarseness** | 6.1 (5.6–6.7) | 5.8 (5.0–6.7) | 14.9 (13.8–16.1) | 12.8 (11.5–14.3) | 13.3 (12.4–14.3) | 14.6 (13.3–15.9) | 8.1 (7.4–8.9) | 7.2 (5.8–8.8) | 6.1 (5.1–7.2) | 5.6 (4.6–6.7) | 7.7 (7.2–8.3) | 6.1 (5.6–6.7) | 6.6 (5.2–8.3) | 4.6 (3.4–6.2) | 7.7 (6.4–9.1) | 7.1 (5.1–9.8) |
| **Suspected cardiac chest pain** | 4.9 (4.4–5.4) | 4.9 (4.2–5.8) | 10.9 (10.0–12.0) | 16.5 (15.0–18.1) | 6.9 (6.2–7.6) | 5.1 (4.3–5.9) | 11.1 (10.2–12.0) | 7.3 (6.0–8.9) | 5.3 (4.4–6.3) | 3.5 (2.8–4.4) | 4.7 (4.3–5.2) | 3.8 (3.4–4.3) | 4.6 (3.5–6.1) | 2.6 (1.7–3.9) | 9.3 (7.9–10.9) | 6.3 (4.4–8.9) |
| **Dysphagia** | 5.2 (4.7–5.8) | 4.6 (3.9–5.4) | 14.8 (13.7–16.0) | 13.1 (11.7–14.6) | 12.5 (11.6–13.4) | 10.9 (9.8–12.1) | 16.7 (15.6–17.7) | 7.2 (5.8–8.8) | 12.5 (11.1–14.0) | 10.6 (9.3–12.1) | 12.1 (11.4–12.8) | 8.3 (7.6–8.9) | 9.5 (7.8–11.4) | 9.3 (7.5–11.3) | 17.3 (15.5–19.3) | 14.6 (11.7–18.1) |
| **Haematemesis** | 0.4 (0.3–0.6) | 0.3 (0.1–0.6) | 0.4 (0.2–0.7) | 0.2 (0.1–0.5) | 0.8 (0.6–1.1) | 0.2 (0.1–0.5) | 1.0 (0.7–1.3) | 0.9 (0.5–1.6) | 2.6 (2.0–3.4) | 0.6 (0.3–1.0) | 0.7 (0.6–0.9) | 0.1 (0.0–0.2) | 1.2 (0.7–2.1) | 0.2 (0.0–0.8) | 1.6 (1.1–2.4) | 0.4 (0.1-1.6) |
| **Black stools** | 9.3 (8.6–10.1) | 7.8 (6.8–8.8) | 10.3 (9.4–11.3) | 7.9 (6.8–9.1) | 11.9 (11.0–12.8) | 8.0 (7.0–9.0) | 14.7 (13.7–15.8) | 8.5 (7.1–10.2) | 10.4 (9.2–11.9) | 4.5 (3.7–5.6) | 7.6 (7.1–8.2) | 5.4 (4.9–5.9) | 6.5 (5.1–8.2) | 4.0 (2.9–5.4) | 10.9 (9.4–12.6) | 11.6 (9.0–14.8) |
| **Haematochezia** | 10.3 (9.6–11.1) | 5.8 (5.0–6.8) | 9.9 (9.0–10.9) | 7.7 (6.6–8.9) | 13.5 (12.6–14.5) | 8.0 (7.0–9.0) | 8.3 (7.5–9.1) | 6.1 (4.9–7.6) | 11.8 (10.5–13.3) | 5.9 (4.9–7.1) | 10.3 (9.7–10.9) | 5.9 (5.4–6.5) | 9.9 (8.3–11.9) | 5.0 (3.7–6.6) | 11.6 (10.1–13.4) | 11.2 (8.6–14.4) |
| **Major change in bowel movement** | 16.3 (15.4–17.2) | 14.3 (13.1–15.6) | 18.8 (17.6–20.1) | 18.0 (16.4–19.6) | 24.5 (23.3–25.7) | 19.0 (17.7–20.5) | 21.2 (20.0–22.4) | 14.9 (13.0–17.0) | 13.9 (12.5–15.5) | 9.2 (8.0–10.6) | 16.0 (15.3–16.8) | 10.7 (10.0–11.4) | 10.6 (8.9–12.6) | 6.7 (5.2–8.5) | 21.4 (19.4–23.5) | 13.4 (10.6–16.8) |
| **Unintentional weight loss** | 2.3 (1.9–2.7) | 1.9 (1.4–2.5) | 3.7 (3.2–4.4) | 2.3 (1.8–3.1) | 4.9 (4.3–5.5) | 2.9 (2.4–3.6) | 5.9 (5.3–6.6) | 2.5 (1.73.5) | 5.7 (4.8–6.9) | 2.0 (1.5–2.8) | 3.0 (2.7–3.4) | 1.4 (1.2–1.8) | 3.8 (2.8–5.2) | 0.9 (0.5–1.8) | 6.7 (5.5–8.1) | 2.8 (1.6–4.8) |
| **Anaemia/low iron** | 17.6 (16.7–18.6) | 11.7 (10.6–12.9) | 14.8 (13.7–15.9) | 8.3 (7.2–9.5) | 9.8 (9.0–10.6) | 5.6 (4.8–6.5) | 22.2 (21.0–23.4) | 12.9 (11.1–14.9) | 17.9 (16.3–19.7) | 12.1 (10.7–13.7) | 11.0 (10.3–11.6) | 6.6 (6.0–7.2) | 17.4 (15.2–19.8) | 9.7 (8.0–11.8) | 17.6 (15.8–19.6) | 9.8 (7.4–12.9) |
| **Fever** | 2.6 (2.2–3.0) | 2.1 (1.6–2.7) | 5.7 (5.0–6.5) | 2.9 (2.2–3.7) | 4.9 (4.3–5.5) | 2.4 (1.9–3.0) | 5.2 (4.6–5.9) | 3.3 (2.4–4.5) | 6.1 (5.1–7.2) | 2.6 (2.0–3.5) | 6.7 (6.2–7.2) | 2.4 (2.1–2.8) | 4.5 (3.3–5.9) | 1.5 (0.8–2.5) | 5.0 (4.0–6.3) | 2.4 (1.3–4.3) |
| **Family history of GI cancer** | 9.9 (9.2–10.7) | 14.5 (13.3–15.9) | 8.3 (7.5–9.2) | 13.6 (12.2–15.1) | 9.9 (9.1–10.7) | 14.8 (13.6–16.1) | 5.6 (5.0–6.3) | 11.1 (9.5–13.0) | 8.8 (7.7–10.1) | 12.1 (10.7–13.7) | 7.0 (6.4–7.5) | 11.0 (10.3–11.8) | 7.7 (6.2–9.5) | 11.0 (9.2–13.2) | 13.3 (11.7–15.2) | 15.2 (12.2–18.8) |
| **Family history of CD** | 2.2 (1.8–2.6) | 2.7 (2.1–3.3) | 3.0 (2.5–3.6) | 2.2 (1.6–2.9) | 5.0 (4.4–5.6) | 3.7 (3.1–4.5) | 2.7 (2.3–3.2) | 2.9 (2.1–4.1) | 3.9 (3.1–4.8) | 2.3 (1.7–3.1) | 4.5 (4.1–5.0) | 1.8 (1.6–2.2) | 4.8 (3.7–6.3) | 2.1 (1.3–3.3) | 2.3 (1.6–3.2) | 1.0 (0.4–2.5) |
| **Family history of IBD** | 6.0 (5.4–6.6) | 7.1 (6.2–8.1) | 3.9 (3.4–4.6) | 5.0 (4.1–6.0) | 8.6 (7.8–9.4) | 7.7 (6.8–8.7) | 7.6 (6.8–8.4) | 6.7 (5.4–8.2) | 8.0 (6.8–9.2) | 4.9 (4.0–6.0) | 4.5 (4.1–5.0) | 3.3 (2.9–3.7) | 4.6 (3.4–6.0) | 3.3 (2.3–4.7) | 9.2 (7.8–10.8) | 3.0 (1.8–5.1) |
| **Cell shading reflects prevalence (%),** ranging from light to dark blue for increasing values (0–3%, 3–5%, 5–10%, 10–15%, 15–20%, >20%). Countries included: Asia; China, Japan, Korea, Singapore; Eastern Europe: Poland, Romania, Russia; Latin America: Argentina, Brazil, Colombia, Mexico; Middle East: Egypt, Israel, Turkey; North America: Canada, United States; Western Europe: Belgium, France, Germany, Holland, Italy, Spain, Sweden. *Abbreviations:* CI, confidence interval; CD, coeliac disease; GI, gastrointestinal; IBD, inflammatory bowel disease; n, number; y, years. | | | | | | | | | | | | | | | | |

| **Supplementary Table 6. Characteristics of participants with and without any self-reported alarm feature** | | |
| --- | --- | --- |
| **Characteristic** | **Any alarm feature (n=31820)** | **No alarm features (n=22229)** |
| Age, mean years (95% CI) | 43  (43–43) | 47  (46–47) |
| Male sex, % (95% CI) | 49.2  (53.6–49.7) | 53.4  (52.7–54.0) |
| Body mass index, mean kg/m^2^ (95% CI) | 25.7  (26–26) | 25.5  (25–26) |
| Unknown, n | 3,146 | 2,604 |
| Education, mean years (95% CI) | 14.1  (14–14) | 14.2  (14–14) |
| Unknown, n | 1,001 | 610 |
| *Abbreviations:* CI, confidence interval; n, number. | | |

| **Supplementary Table 7. Prevalence of individual self-reported alarm features among participants with and without DGBI, by age group** | | | | | | |
| --- | --- | --- | --- | --- | --- | --- |
|  | **Age <50 years** | | | **Age ≥50 years** | | |
| **Alarm features** | **No DGBI, n**=**17,996**  **% (95% CI)** | **Any DGBI, n**=**14,174**  **% (95% CI)** | **OR (95% CI)** | **No DGBI, n**=**11,370**  **% (95% CI)** | **Any DGBI, n**=**6,494**  **% (95% CI)** | **OR (95% CI)** |
| Dysphagia | 7.9 (7.5–8.3) | 15.6 (15.0–16.2) | **2.2 (2.0–2.3)** | 5.0 (4.6–5.4) | 13.9 (13.0–14.7) | **3.1 (2.8–3.4)** |
| Neck/throat pain | 9.6 (9.2–10.0) | 18.8 (18.2–19.5) | **2.2 (2.0–2.3)** | 5.0 (4.6–5.4) | 12.1 (11.3–12.9) | **2.6 (2.3–2.9)** |
| Hoarseness | 6.4 (6.1–6.8) | 11.1 (10.5–11.6) | **1.8 (1.7–2.0)** | 5.6 (5.2–6.0) | 11.0 (10.3–11.8) | **2.1 (1.9–2.3)** |
| Suspected cardiac chest pain | 3.6 (3.4–3.9) | 9.7 (9.2–10.2) | **2.9 (2.6–3.1)** | 2.8 (2.5–3.1) | 9.3 (8.6–10.1) | **3.6 (3.1–4.1)** |
| Haematemesis | 0.3 (0.2–0.4) | 1.2 (1.1–1.4) | **4.7 (3.5–6.6)** | 0.1 (0.0–0.2) | 0.4 (0.3–0.6) | **4.9 (2.4–11)** |
| Black stools | 5.9 (5.6–6.3) | 14.4 (13.9–15.0) | **2.7 (2.5–2.9)** | 3.4 (3.1–3.8) | 10.6 (9.8–11.4) | **3.3 (2.9–3.8)** |
| Haematochezia | 6.5 (6.1–6.8) | 14.7 (14.1–15.3) | **2.5 (2.3–2.7)** | 3.5 (3.1–3.8) | 10.5 (9.8–11.3) | **3.3 (2.9–3.7)** |
| Major change in bowel movements | 10.5 (10.1–11.0) | 26.4 (25.7–27.1) | **3.0 (2.9–3.2)** | 7.3 (6.8–7.8) | 20.8 (19.8–21.8) | **3.3 (3.0–3.7)** |
| Unintentional weight loss | 2.3 (2.1–2.5) | 5.2 (4.8–5.6) | **2.3 (2.1–2.6)** | 1.0 (0.9–1.3) | 2.7 (2.3–3.1) | **2.6 (2.1–3.3)** |
| Anaemia/low iron * | 10.2 (9.8–10.7) | 19.4 (18.8–20.1) | **2.1 (2.0–2.3)** | 5.5 (5.1–5.9) | 11.6 (10.8–12.4) | **2.3 (2.0–2.5)** |
| Fever | 3.6 (3.3–3.8) | 6.3 (5.9–6.7) | **1.8 (1.6–2.0)** | 1.7 (1.5–2.0) | 3.0 (2.6–3.4) | **1.8 (1.4–2.1)** |
| Family history of GI cancer | 6.4 (6.0–6.7) | 9.6 (9.1–10.1) | **1.6 (1.4–1.7)** | 10.2 (9.6–10.8) | 14.7 (13.8–15.6) | **1.5 (1.4–1.7)** |
| Family history of coeliac disease | 2.7 (2.4–2.9) | 3.4 (3.2–3.8) | **1.3 (1.1–1.5)** | 1.6 (1.4–1.9) | 2.6 (2.3–3.1) | **1.7 (1.3–2.0)** |
| Family history of IBD | 3.6 (3.3–3.9) | 7.0 (6.6–7.4) | **2.0 (1.8–2.2)** | 2.9 (2.6–3.2) | 6.0 (5.5–6.6) | **2.2 (1.9–2.5)** |
| * Sex-stratified prevalence: <50 years, female 25.9% vs 16.8% (OR 1.7, 95% CI 1.6-1.9), male 10.1% vs 4.6% (2.3, 95% CI 2.0-2.6); **≥50 years, female 14.2% vs 7.3% (OR 2.1, 95% CI 1.8-2.4), male 8.6% vs 4.3% (OR 2.1, 95% CI 1.8-2.5).** OR are unadjusted and should be interpreted as descriptive measures of between-group differences. OR values with 95% CI not including 1.0 are shown in bold. Participants with a reported history of organic or structural gastrointestinal disease were excluded from the analyses. Self-reported alarm features include the 14 items (symptoms, signs, and family history) appended to the Rome IV Diagnostic Questionnaire for adult DGBI. *Abbreviations:* CI, confidence interval; DGBI, disorder of gut-brain interaction; GI, gastrointestinal; IBD, inflammatory bowel disease; n, number; OR, odds ratio. | | | | | | |

| **Supplementary Table 8. Regional prevalence of any self-reported alarm feature by DGBI status and age group** | | | | | | | | | | |
| --- | --- | --- | --- | --- | --- | --- | --- | --- | --- | --- |
| **Region** | **Age <50 years** | | | | | **Age ≥50 years** | | | | |
|  | **No DGBI** | | **Any DGBI** | | **OR (95% CI)** | **No DGBI** | | **Any DGBI** | | **OR (95% CI)** |
|  | **n** | **% (95% CI)** | **n** | **% (95% CI)** |  | **n** | **% (95% CI)** | **n** | **% (95% CI)** |  |
| Asia | 3,779 | 48.8 (47.2–50.4) | 2,333 | 68.7 (66.7–70.5) | **2.3 (2.1–2.6)** | 1,782 | 37.8 (35.6–40.1) | 850 | 66.4 (63.0–69.5) | **3.2 (2.7–3.9)** |
| **Eastern Europe** | 2,040 | 57.7 (55.5–59.9) | 1,697 | 78.7 (76.7–80.6) | **2.7 (2.3–3.1)** | 1,179 | 51.1 (48.3–54.0) | 849 | 78.9 (76.0–81.6) | **3.6 (2.9–4.4)** |
| **Latin America** | 2,468 | 58.1 (56.2–60.1) | 2,309 | 83.0 (81.3–84.5) | **3.5 (3.1–4.0)** | 1,668 | 47.9 (45.5–50.3) | 1,026 | 78.1 (75.4–80.6) | **3.9 (3.3–4.6)** |
| **Middle East** | 2,570 | 60.1% (58.2–62.0) | 1,914 | 82.7 (80.9–84.4) | **3.2 (2.8–3.7)** | 714 | 47.1 (43.4–50.8) | 442 | 70.4 (65.8–74.5) | **2.7 (2.1–3.4)** |
| **North America** | 1,056 | 40.6 (37.7–43.7) | 906 | 72.5 (69.4–75.3) | **3.8 (3.2–4.7)** | 1,071 | 32.2 (29.4–35.1) | 664 | 62.8 (59.0–66.5) | **3.6 (2.9–4.4)** |
| **Western Europe** | 4,738 | 43.5 (42.1–44.9) | 3,913 | 70.5 (69.1–71.9) | **3.1 (2.8–3.4)** | 4,161 | 33.2 (31.8–34.7) | 2,197 | 60.8 (58.7–62.8) | **3.1 (2.8–3.5)** |
| **Australia** | 582 | 39.4  (35.4–43.5) | 447 | 64.4  (59.8–68.8) | **2.8**  **(2.2–3.6)** | 533 | 28.9  (25.1–33.0) | 284 | 54.2  (48.2–60.1) | **2.9**  **(2.2–3.9)** |
| **South Africa** | 762 | 54.0  (50.4–57.6) | 655 | 80.6  (77.3–83.5) | **3.5**  **(2.8–4.5)** | 262 | 44.3  (38.2–50.5) | 182 | 73.1  (65.9–79.2) | **3.4**  **(2.3–5.2)** |
| OR are unadjusted and should be interpreted as descriptive measures of between-group differences. OR values with 95% CI not including 1.0 are shown in bold. Participants with a reported history of organic or structural gastrointestinal disease were excluded from the analyses. Self-reported alarm features include the 14 items (symptoms, signs, and family history) assessed in the Rome IV Diagnostic Questionnaire for adult DGBI. *Abbreviations:* CI, confidence interval; DGBI, disorder of gut-brain interaction; n, number; OR, odds ratio. | | | | | | | | | | |

| **Supplementary Table 9. Prevalence and number of self-reported traditional gastrointestinal alarm features among individuals with and without DGBI, by age group** | | | | | | |
| --- | --- | --- | --- | --- | --- | --- |
|  | **Age <50 years** | | | **Age ≥50 years** | | |
| **Number of self-reported alarm features** | **Any DGBI**  **(n=14,174)** | **No DGBI**  **(n=17,995)** | **OR**  **(95% CI)** | **Any DGBI**  **(n=6,494)** | **No DGBI**  **(n=11,370)** | **OR**  **(95% CI)** |
| Any, % (95% CI) | 54.1  (53.3–54.9) | 31.9  (31.2–32.6) | **2.5**  **(2.4–2.6)** | 46.6  (45.4–47.8) | 24.2  (23.4–25.0) | **2.7**  **(2.6–2.9)** |
| **1,** % (95% CI) | 34.8  (34.0–35.6) | 25.6  (24.9–26.2) | **1.6**  **(1.5–1.6)** | 33.2  (32.1–34.4) | 20.3  (19.5–21.0) | **2.0**  **(1.8–2.1)** |
| **≥2,** % (95% CI) | 19.3  (18.6–20.0) | 6.4  (6.0–6.7) | **3.5**  **(3.3–3.8)** | 13.4  (12.6–14.3) | 3.9  (3.6–4.3) | **3.8**  **(3.3–4.3)** |
| **≥3,** % (95% CI) | 5.2  (4.8–5.6) | 0.9  (0.8–1.1) | **6.0**  **(5.1–7.1)** | 3.4  (3.0–3.9) | 0.4  (0.3–0.6) | **8.3**  **(6.1–11.4)** |
| Mean (95% CI) | 0.8  (0.8–0.8) | 0.4  (0.4–0.4) | N/A | 0.6  (0.6–0.7) | 0.3  (0.3–0.3) | N/A |
| OR are unadjusted and should be interpreted as descriptive measures of between-group differences. OR values with 95% CI not including 1.0 are shown in bold. Participants with a reported history of organic or structural gastrointestinal disease were excluded from the analyses. Self-reported alarm features include the items on dysphagia, haematemesis, black stools, haematochezia, anaemia/low iron, unintentional weight loss, and family history of gastrointestinal cancer appended to in the Rome IV Diagnostic Questionnaire for adult disorders of gut-brain interaction. *Abbreviations:* CI, confidence interval; DGBI, disorder of gut-brain interaction; n, number; OR, odds ratio. | | | | | | |

| **Supplementary Table 10. Prevalence of any and individual self-reported alarm features by age group in individuals with a self-reported history of organic gastrointestinal disease without concurrent disorders of gut-brain interaction** | | |
| --- | --- | --- |
| **Self-reported alarm features** | **Age <50 years**  **(n=1,351)** | **Age ≥50 years**  **(n=1,698)** |
|  | **% (95% CI)** | **% (95% CI)** |
| Any | 88.4  (86.6–90.1) | 67.5  (65.2–69.7) |
| Neck/throat pain | 23.8  (21.5–26.1) | 12.5  (11.0–14.2) |
| Hoarseness | 16.7  (14.8–18.9) | 10.9  (9.5–12.5) |
| Suspected cardiac chest pain | 15.2  (13.4–17.3) | 8.4  (7.2–9.9) |
| Dysphagia | 22.3  (20.1–24.6) | 12.5  (11.0–14.2) |
| Haematemesis | 3.8  (2.9–5.0) | 0.8  (0.4–1.3) |
| Black stools | 16.8  (14.9–18.9) | 9.9  (8.5–11.4) |
| Haematochezia | 20.3  (18.2–22.5) | 9.1  (7.8–10.6) |
| Major change in bowel movements | 29.2  (26.8–31.8) | 19.0  (17.1–20.9) |
| Unintentional weight loss | 11.9  (10.3–13.8) | 4.0  (3.1–5.1) |
| Anaemia/low iron | 26.6  (24.3–29.0) | 14.5  (12.9–16.3) |
| Fever | 11.6  (10.0–13.5) | 4.4  (3.5–5.5) |
| Family history of gastrointestinal cancer | 18.1  (16.1–20.2) | 18.4  (16.6–20.4) |
| Family history of coeliac disease | 16.1  (14.2–18.2) | 6.3  (5.2–7.6) |
| Family history of inflammatory bowel disease | 26.9  (24.5–29.3) | 13.8  (12.3–15.6) |
| Self-reported organic gastrointestinal diseases included: ulcerative colitis, Crohn’s disease, coeliac disease, gastrointestinal cancer, peptic ulcer disease, diverticulitis, prior bowel resection. *Abbreviations:* CI, confidence interval; n, number. | | |
